# NF-kB affects migration of vascular smooth muscle cells after treatment with heparin and ibrutinib

**DOI:** 10.1016/j.bbrep.2024.101685

**Published:** 2024-03-16

**Authors:** Nafiseh Shokri, Ghasem Ghasempour, Ali Akbar Soleimani, Mohammad Elahimanesh, Mohammad Najafi

**Affiliations:** a Clinical Biochemistry Department, Faculty of Medical Sciences, Iran University of Medical Sciences, Tehran, Iran; b Davee Department of Neurology, Feinberg School of Medicine, Northwestern University, Chicago, Illnosis, USA; c Clinical Biochemistry Department, Faculty of Medical Sciences, Tehran University of Medical Sciences, Tehran, Iran; d Microbial Biotechnology Research Center, Iran University of Medical Sciences, Tehran, Iran; e Clinical Biochemistry Department, Faculty of Medicine, Shahid Beheshti University of Medical Sciences, Tehran, Iran

**Keywords:** VSMCs, Heparin, NF-kB, Collagen, Betulinic acid, Ibrutinib

## Abstract

The migration of vascular smooth muscle cells (VSMCs) is one of the most important events in the remodeling of atherosclerosis plaque. The aim of study was to investigate the role of Heparin in the VSMC migration and its association with the NF-kB, collagen 1 and collagen 3 expression levels. Moreover, the incorporation of Heparin was studied in the VSMC cultures including Betulinic acid and Ibrutinib. Twelve cell groups were cultured and treated with the Heparin, Betulinic acid and Ibrutinib based on the viability and toxicity in 24-h and 48-h periods. The gene and protein expression levels were measured by RT-qPCR and western blotting techniques. The VSMC migration was determined by scratch test. In contrast with Ibrutinib (2 μM), Heparin (30 IU) increased significantly (P < 0.05) the NF-kB gene and protein expression levels and the VSMC migration during the exposure periods. Heparin (15 IU and 30 IU) also increased the collagen 1 gene expression level in the 48-h period while Heparin (5 IU and 15 IU) increased the collagen 3 gene expression levels in both periods. Incorporating Heparin into the cultures including Betulinic acid and Ibrutinib affected the collagen 1 and collagen 3 expression levels. The data suggested that the cell migration relates to NF-kB in the VSMCs treated with Heparin and Ibrutinib. Furthermore, the Heparin doses (5 IU and 15 IU) were safe for VSMCs based on the NF-kB, and collagen 3 expression levels.

## Highlights

• Heparin affected the collagen 1 and collagen 3 and NF-KB gene expression levels.
• Ibrutinib decreased the collagen 1 and collagen 3 and NF-KB gene expression levels.
• Heparin and Ibrutinib affected the migration of VSMCs.

## 1 Introduction

Atherosclerosis is known as a vascular disorder due to the formation of atheromatous plaques. The plaques appear and grow by remodeling fatty compounds, cells, and other extracellular substances within the vessel sub-endothelial space [1]. It is critical to realize the molecular and cellular events of atherosclerosis process. Studies have shown that macrophages, endothelial cells, and smooth muscle cells (SMCs) are involved in the progression of atherosclerotic lesions [2]. Although the vascular smooth muscle cells (VSMCs) are crucial for the good performance of the vasculature [3] but their phenotype changes based on the vessel microenvironment play a vital role in the secretion of extracellular matrix (ECM), and finally the advancement of lesion [4]. Whereas ECM constitutes most collagen 1 and 3 in healthy arteries, the atherosclerosis lesions mainly contain collagen 1 and fibronectin [4]. Furthermore, the role of VSMCs is reported to contribute in the plaque rupture [5]. Some studies reported the involved VSMC signaling pathways during the progression of atherosclerosis [6]. It is reported that Nuclear factor kappa-B (NF-kB) via modulation of some genes affects inflammatory events [[7], [8], [9]] so the suppression of these events prevents the progression of vessel stenosis [10]. Heparin is reported to hinder the cellular growth via the TNF and NF-kB signaling axes. It may inhibit the roles of adhesion molecules, most notably P-Selectin and L-Selectin, involved in the inflammatory reactions [[11], [12], [13]]. Some compounds and drugs are also involved in these cellular signaling pathways. As example, Betulinic acid affected the cellular growth via the involved pathways with NF-kB [14,15]. It might mediate the activation of NF-kB axis by changing IKK activity. NF-kB also might modify the extracellular matrix (ECM) formation via matrix metalloproteinase (MMP) activity and collagen fibers [16]. More studies on the functions of Betulinic acid in signaling pathways investigated in some cancerous cells [9]. In addition, Ibrutinib as the BTK (Bruton Tyrosine Kinase) inhibitor suppressed NF-kB in CLL cells [17]. Ibrutinib was also reported to suppress the cellular proliferation and apoptosis [18,19].

The aim of this study was to investigate the effect of Heparin on the migration of vascular smooth muscle cells and its relationship with the collagen 1 (COL1A1), collagen 3 (COL3A1) and NF-kB expression levels. Furthermore, the above reports suggested that Betulinic acid improves cellular growth while some reports showed that Ibrutinib suppresses cellular proliferation. Thus, these compounds were applied in this study to compare and discuss results obtained from the role of Heparin.

## 2 Material and methods

### 2.1 Cell culture

The vascular smooth muscle cells (NCBI Code, C591) were obtained from Pasteur Institute (Tehran). The cells were cultured in Dulbecco's modified eagle medium (DMEM-F12, Cat. No. BI-1011, Bioidea, Iran) by adding 10% Fetal Bovine Serum (FBS, Cat. No. BI-1201, Bioidea, Iran) and 1% Penicillin–Streptomycin (Cat. No BI-1203, Bioidea, Iran).

### 2.2 Cell viability

The cellular viability was determined for Heparin, Betulinic acid (EC. No. 207-488-6), and Ibrutinib (CAS NO: 936563-96-1). The MTT assay was used to measure the cellular viability and cytotoxicity [20]. The VSMCs (4 × 10^3^ cells) were seeded in 96-well plates and treated with the different values of Heparin (0–500 IU/ml), Betulinic acid (18–60 μM), and Ibrutinib (0.2–7.4 μM) for 24 and 48 h. After removing the medium, the cells were treated and incubated with MTT (0.5 mg/ml, Cat. No. M5655, Sigma, USA) solution for 4 h at 37 °C. Then, DMSO (200 μL) was added to dissolve the crystals, and the mixture was agitated (15 min, room temperature). Finally, OD was calculated at a wavelength of 570 nm. Three doses (5, 15 and 30 IU; Cell viability >50%) for Heparin, dose 60 μM for Betulinic acid (Cell viability >90%), and dose 2 μM for Ibrutinib (Cell viability >60%) were applied in this study. Twelve cellular groups including Control, Heparin (5, 15 and 30 IU), Betulinic acid (60 μM), Betulinic acid (60 μM) (+Heparin; (5, 15 and 30 IU)), Ibrutinib (2 μM), and Ibrutinib (2 μM) (+Heparin; (5, 15 and 30 IU)) were studied in 24-h and 48-h periods.

### 2.3 RNA extraction, cDNA synthesis and RT-qPCR

By using of RNA extraction kit (Cat. No. EX60311, Sinacolon, Iran), total RNA was isolated from all the cellular groups in the 24-h and 48-h periods. According to the producer's instructions, the cell plate was lysed using the lysis buffer, and the supernatant was eluted and washed on a column using alcoholic gradient solutions. The RNA quantity and quality were evaluated by Nanodrop. The cDNA synthesis was carried out with Yektatajhiz kit (Iran). The RNA samples were incubated with the RTase, random primers, RNAase inhibitor and, dNTPs for 75 min at 37 °C. SYBR Green PCR Master Mix (Cat. No. YT2551, Yektatajhiz, Iran) was used for the RT-qPCR reaction. The cDNA samples were amplified with specific primers during 40 cycles using annealing temperatures (NF-kB, 58 °C; COL1A1, 57 °C; COL3A1, 60 °C; GAPDH, 58 °C). The GAPDH gene was applied as a gene reference. The gene primers are reported in Table 1.

**Table 1:** The primers.

Gene	Forward sequence	Reverse sequence
**NF-kB**	5′- GGGGACTACGACCTGAATGC -3′	5′-ACACCTCAATGTCCTCTTTCTGC- 3′
**COL1A1**	5′-AAGACATCCCACCAATCACC-3′	5′-GGCAGTTCTTGGTCTCGTCA -3′
**COL3A1**	5′-GACTTCTCTCCAGCCGAGC-3′	5′-TTTTGCTCCATTCCCCAGTGT-3′
**GAPDH**	5′-CATGAGAAGTATGACAACAGCCT-3′	5′-AGTCCTTCCACGATACCAAAGT-3′

### 2.4 Western blotting

The cells in all the groups were separated from the flasks with a scraper. Then, total protein was extracted with RIPA buffer (Cat. No. DB9719, DNA biotech, Iran) containing protease inhibitors and phenylmethyl sulfonyl fluoride (PMSF), shaking the cell plate for 5 min. The solution was centrifuged at 13000g for 20 min at 4 °C. Then, the supernatant sample was run on SDS-PAGE (12%) gel for 45 min (86V). The protein contents were transferred to PVDF (Cat. No. IPVH00010, Merck Millipore, Germany) for 60 min. The PVDF sheets were blocked with milk (4%, Cell Signaling Technology, USA) for 60 min, before being incubated overnight at 4 °C with a primary NF-kB antibody (1:500 v/v, Cat. No. sc7151, Santa Cruz, California, USA). Subsequent washing in tris-buffered saline containing 0.1% Tween 20 (TBST), the PVDF membrane was incubated for 60 min at room temperature with a secondary antibody (1:2000 v/v, Cat. No. 7074s, Cell Signaling Technology, Beverly, MA, USA). Then, the membrane was incubated with enhanced chemiluminescence (ECL). The protein values were adjusted using beta-Actin (1:1000 v/v, Cat. No. 4967s, Cell Signaling Technology, Beverly, MA, USA).

### 2.5 Scratch test

Cellular migration was evaluated using the scratch test [21]. The VSMCs were cultured in a 6-well plate for reaching to appropriate confluency (70%) in order to harvest with a pipette tip. After washing with PBS (Cat. No. BCBS2233V, Sigma, Germany), the treated cell groups were cultured for the 24-h and 48-h periods. An inverted microscope was used to obtain the images of harvested plates.

### 2.6 Statistical analysis

The data were analyzed using GraphPad Prism (Version 8.0.3). The Kolmogorov-Simonov test was initially used to evaluate data distribution. The One-Way ANOVA and Tukey tests were applied to compare the data between the cell groups. Furthermore, the student's t-test was applied to compare two independent groups. To determine the changes in gene expression levels, the 2^−ΔΔCT^ formula was used. For the Western blot and scratch image analyses, Image J software was used.

## 3 Results

### 3.1 Viability and cytotoxicity

**Fig. 1:**
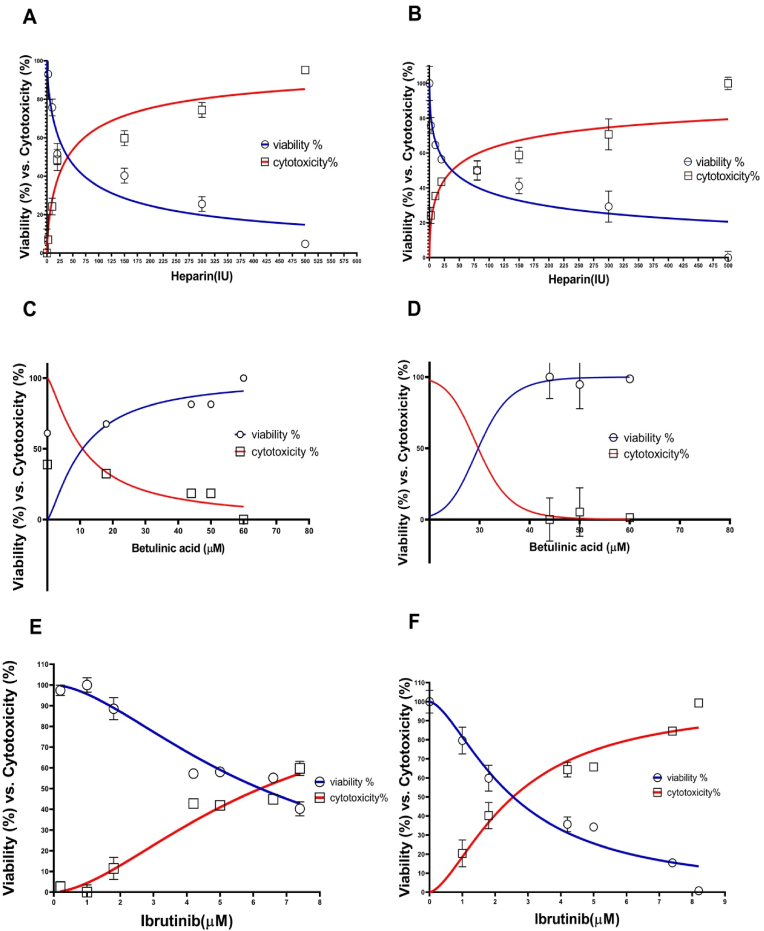
The effects of Heparin, Betulinic acid, and Ibrutinib on VSMC viability. The cell viability and cytotoxicity were estimated in 24-h and 48-h periods. **A, C, E;** 24-h period. **B, D, F**; 48-h period.

The viability and cytotoxicity of vascular smooth muscle cells were evaluated on the concentration gradients of Heparin, Betulinic acid and Ibrutinib. The IC50 values for Heparin, and Ibrutinib were estimated 40.45 IU, and 6.2 μM for the 24-h period, and also 37.95 IU, and 2.5 μM for the 48-h period, respectively. However, Betulinic acid improved the cellular viability in both periods (Fig. 1, A-F).

### 3.2 Gene expression levels

#### 3.2.1 NF-kB gene expression levels

##### 3.2.1.1 24-H period

**Fig. 2:**
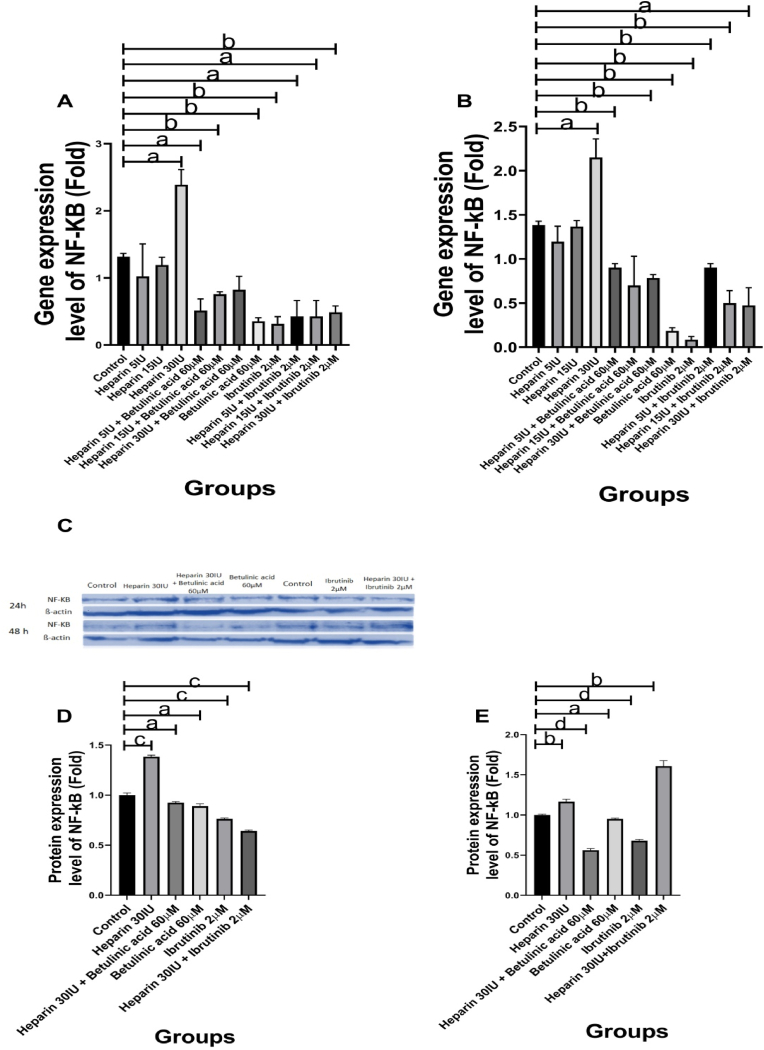
The NF-kB gene and protein expression levels of vascular smooth muscle cells (VSMCs) in the studied groups. **Gene expression levels, A: 24-h period,** In contrast with the Betulinic acid 60 μM and Ibrutinib 2 μM treated groups, the NF-kB gene expression levels increased significantly in Heparin 30 IU treated group. **B: 48-h period,** The NF-kB gene expression levels in the Heparin 30 IU treated group increased significantly while it reduced in Betulinic acid 60 μM, and Ibrutinib 2 μM treated groups. **Protein expression levels, C:** Western blot image. The image is cropped from the original picture as presented in Supplement 1. **D: 24-h period,** Heparin 30 IU increased significantly NF-kB protein expression level but Betulinic acid 60 μM and Ibrutinib 2 μM reduced it. **E: 48-h period,** The results were similar to the 24-h period but Heparin 30 IU and Heparin 30 IU + Ibrutinib 2 μM increased significantly NF-kB protein expression levels. All cellular groups are compared to control. a, p < 0.05, b and c, p < 0.0001; d, p < 0.001.

The NF-kB gene expression levels increased significantly in higher values (30 IU) of Heparin (P = 0.0233) as compared with lower values of Heparin 5 IU (P = 0.4844), and 15 IU (P = 0.3178). In contrast with the Heparin (30 IU) results, the NF-kB gene expression levels reduced in the Betulinic acid (P = 0.0039) and Ibrutinib (P = 0.0079) treated groups. The NF-kB gene expression levels in the cellular groups containing Betulinic acid and Heparin were compared with the Betulinic acid-treated group. The results showed that Heparin does not affect the NF-kB gene expression level at 5 IU dose (Betulinic acid + Heparin 5 IU, P = 0.3391). At the same time, it increased the NF-kB gene expression levels at 15 and 30 IU doses (Betulinic acid (+Heparin: 15 IU, P = 0.013; 30 IU, P = 0.049)). Furthermore, the NF-kB gene expression levels in the cellular groups containing Ibrutinib and Heparin were compared with the Ibrutinib-treated group. The data showed that Heparin does not affect the NF-kB gene expression levels (Ibrutinib (+Heparin: 5 IU, P = 0.6113; 15 IU, P = 0.5812; 30 IU, P = 0.2330)) (Fig. 2, A).

##### 3.2.1.2 48-H period

Similar to the 24-h period results, the NF-kB gene expression levels increased significantly in Heparin 30 IU group (P = 0.0372) while in the groups of 5 IU (P = 0.2824) and 15 IU (P = 0.8148) were not significant. In contrast with the Heparin (30 IU) results, the NF-kB gene expression levels reduced in the Betulinic acid (P = 0.0012), and Ibrutinib (P = 0.0010) treated groups. The NF-kB gene expression levels after incorporating Heparin (Betulinic acid + Heparin) were compared with the Betulinic acid-treated group. The results showed that Heparin increases the NF-kB gene expression levels (Betulinic acid (+Heparin: 5 IU, P = 0.0031; 15 IU, P = 0.04812; 30 IU, P = 0.0038)). Furthermore, the NF-kB gene expression levels in the cellular groups containing Ibrutinib and Heparin were compared with the Ibrutinib-treated group. The data showed that Heparin increases the NF-kB gene expression levels (Ibrutinib (+Heparin: 5 IU, P = 0.0024; 15 IU, P = 0.04912)). However, the NF-kB gene expression level did not change at 30 IU dose from Heparin (Ibrutinib + Heparin 30 IU, P = 0.0658) (Fig. 2, B).

#### 3.2.2 Collagen 1 gene expression levels

##### 3.2.2.1 24-H period

**Fig. 3:**
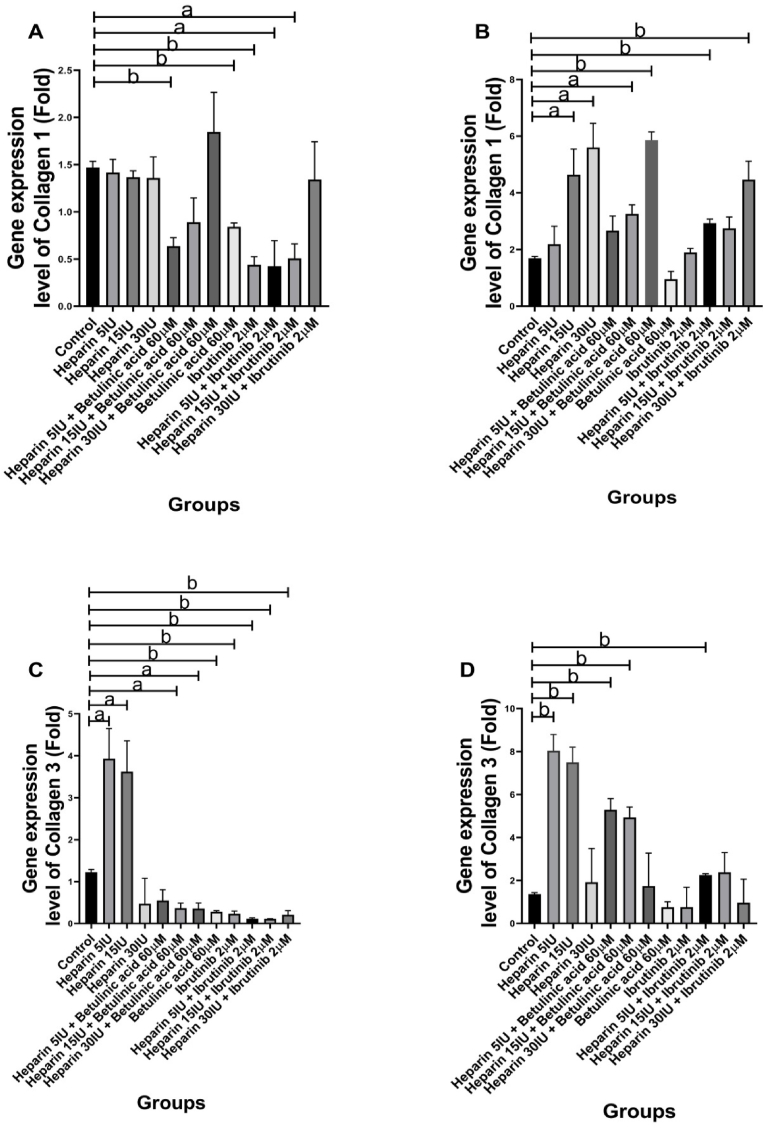
Collagen 1 and Collagen 3 gene expression levels of vascular smooth muscle cells (VSMCs) in the studied groups. **Collagen 1 expression levels, A: 24-h period:** Betulinic acid 60 μM and Ibrutinib 2 μM reduced significantly collagen 1 expression levels. **B**: **48-h period:** The changes of collagen 1 expression levels did not relate significantly to Betulinic acid 60 μM and Ibrutinib 2 μM. **Collagen 3 expression levels, C: 24-h period:** Collagen 3 expression levels were significantly increased in the Heparin 5 IU and 15 IU treated groups. **D: 48-h period:** Similar to the 24-h period, the collagen 3 expression levels were significantly increased in Heparin 5 IU and 15 IU treated groups. All cellular groups are compared to control. a, p < 0.05; b, p < 0.002.

The collagen 1 gene expression levels did not change significantly in Heparin (5 IU (P = 0.6905), 15 IU (P = 0.2724), and 30 IU (P = 0.5735)) treated groups. However, it decreased significantly in the Betulinic acid (P = 0.0079) and Ibrutinib (P = 0.0056) treated groups. The collagen 1 gene expression levels in the cellular groups containing Betulinic acid and Heparin were compared with the Betulinic acid-treated group. The data showed that Heparin does not affect the collagen 1 gene expression levels (Betulinic acid (+Heparin: 5 IU, P = 0.1380; 15 IU, P = 0.8113)). However, it increased the collagen 1 gene expression levels at 30 IU (Betulinic acid + Heparin 30 IU, P = 0.046). Furthermore, the collagen 1 gene expression levels in the cellular groups containing Ibrutinib and Heparin were compared with the Ibrutinib-treated group. The data showed that Heparin does not affect the collagen 1 gene expression levels (Ibrutinib (+Heparin: 5 IU, P = 0.9470; 15 IU, P = 0.6360)). However, the Heparin increased the collagen 1 gene expression level at 30 IU dose (Ibrutinib + Heparin 30 IU, P = 0.05) (Fig. 3, A).

##### 3.2.2.2 48-H period

The collagen 1 gene expression levels increased significantly in Heparin 15 IU (P = 0.0442), and 30 IU (P = 0.0229) treated groups while it had no change in Betulinic acid- and Ibrutinib-treated groups. The collagen 1 gene expression levels in the cellular groups after incorporating Heparin (Betulinic acid + Heparin) were compared with the Betulinic acid-treated group. The data showed that Heparin increases the collagen 1 gene expression levels (Betulinic acid (+Heparin: 5 IU, P = 0.05; 15 IU, P = 0.0164; 30 IU, P = 0.0033)). Furthermore, the collagen 1 gene expression levels in the cellular groups containing Ibrutinib and Heparin were compared with the Ibrutinib-treated group. The data showed that Heparin increases the collagen 1 gene expression levels (Ibrutinib (+Heparin: 5 IU, P = 0.0186; 15 IU, P = 0.0469; 30 IU, P = 0.0032)) (Fig. 3, B).

#### 3.2.3 Collagen 3 gene expression levels

##### 3.2.3.1 24-H period

The collagen 3 gene expression levels increased significantly in Heparin 5 IU (P = 0.0339), and 15 IU (P = 0.0444) treated groups but it had not the significant change in Heparin 30 IU (P = 0.2236) treated group. It decreased in the Betulinic acid (P = 0.0027) and Ibrutinib (P = 0.0043) treated groups. The collagen 3 gene expression levels in the cellular groups containing Betulinic acid and Heparin were compared with the Betulinic acid-treated group. The results showed that Heparin does not affect the collagen 3 gene expression levels (Betulinic acid (+Heparin: 5 IU, P = 0.2788; 15 IU, P = 0.4349; 30 IU, P = 0.5153)). Furthermore, the collagen 3 gene expression levels in the cellular groups containing Ibrutinib and Heparin were compared with the Ibrutinib-treated group. The data showed that Heparin does not affect the collagen 3 gene expression levels (Ibrutinib (+Heparin; 5 IU, P = 0.1480; 15 IU, P = 0.1320; 30 IU, P = 0.8222) (Fig. 3, C).

##### 3.2.3.2 48-H period

Similar to the 24-h period, the collagen 3 gene expression levels increased significantly in Heparin 5 IU (P = 0.0065), and 15 IU (P = 0.0066) treated groups while it did not change in the Betulinic acid (P = 0.27), and Ibrutinib (P = 0.4524) treated groups. The collagen 3 gene expression levels in the cellular groups after incorporating Heparin (Betulinic acid + Heparin) were compared with the Betulinic acid-treated group. The results showed that Heparin increased the collagen 3 gene expression levels (Betulinic acid (+Heparin: 5 IU, P = 0.0080; 15 IU, P = 0.0084)). However, it did not affect the collagen 3 gene expression level at 30 IU (Betulinic acid + Heparin 30 IU, P = 0.4626). Furthermore, the collagen 3 gene expression levels in the cellular groups containing Ibrutinib and Heparin were compared with the Ibrutinib-treated group. The data showed that Heparin does not affect the collagen 3 gene expression levels (Ibrutinib (+Heparin: 5 IU, P = 0.1510; 15 IU, P = 0.2187; 30 IU, P = 0.8593)) (Fig. 3, D).

### 3.3 NF-kB protein expression levels

#### 3.3.1 24-H period

The NF-kB protein expression level increased significantly (P = 0.0001) in Heparin 30 IU treated group. The Betulinic acid 60 μM (P = 0.0266), Ibrutinib 2 μM (P = 0.0005), Heparin 30 IU + Betulinic acid 60 μM (P = 0.0312) and Heparin 30 IU + Ibrutinib 2 μM (P = 0.0001) treated groups reduced significantly the NF-kB protein expression levels as compared to the control group (Fig. 2, C and D).

#### 3.3.2 48-H period

Similar to the 24-h period, the NF-kB protein expression levels increased significantly in Heparin 30 IU (P = 0.0078) treated group while in other groups including Betulinic acid 60 μM (P = 0.0255), Ibrutinib 2 μM (P = 0.0001), Heparin 30 IU + Betulinic acid 60 μM (P = 0.0001) reduced significantly as compared to the control group. Furthermore, there was a significant increase in Heparin 30 IU + Ibrutinib 2 μM treated group (P = 0.001) (Fig. 2, C and E).

### 3.4 Scratch assay

#### 3.4.1 24-H period

**Fig. 4:**
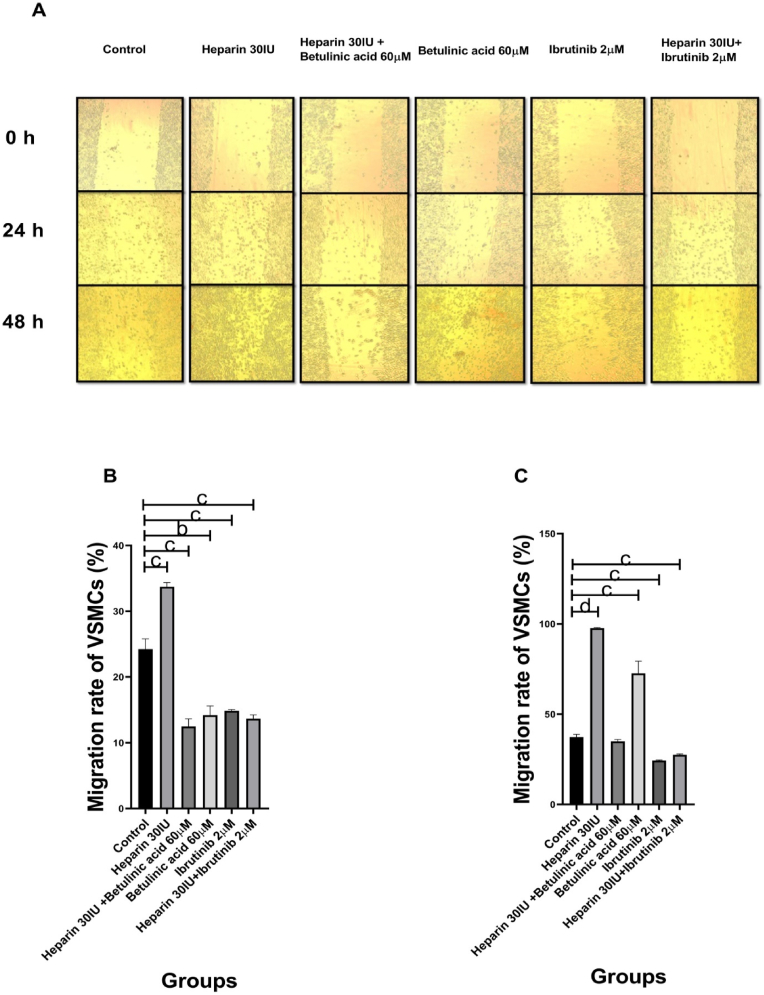
The effects of Heparin, Betulinic acid, and Ibrutinib on the migration of VSMCs. **A**: Images of scratch assay technique. **B: 24-h period**: Heparin 30 IU increased significantly the VSMC migration. **C: 48-h period**. The Heparin 30 IU and Betulinic acid 60 μM increased significantly the cellular migration. All cellular groups are compared to control. b:p < 0.002, c:p < 0.0002, d:p < 0.0001.

The cellular migration increased significantly in the Heparin 30 IU treated group (P = 0006). In contrast with the above group, the cellular migration decreased in other groups including the Betulinic acid 60 μM (P = 0.0012), Ibrutinib 2 μM (P = 0.0048), Heparin 30 IU + Betulinic acid 60 μM (P = 0.005), Heparin 30 IU + Ibrutinib 2 μM (P = 0.0004) groups as compared to the control group (Fig. 4, A and B).

#### 3.4.2 48-H period

The cellular migration increased significantly in the Heparin 30 IU (P = 0.0001), and Betulinic acid 60 μM (P = 0.0009) treated groups. However, the cellular migration decreased in Ibrutinib 2 μM (P = 0.0001), Heparin 30 IU + Ibrutinib 2 μM (P = 0.0004) treated groups as compared to the control group (Fig. 4, A and C).

## 4 Discussion

Atherosclerosis is a disorder in which atherogenic deposits cause vessel stenosis. Many reports showed that lifestyle, genetics and environmental agents might develop the atherosclerosis process. However, the studies of cellular functional changes in the vessel sub-endothelial space are the most important essential subjects for understanding the mechanism of atherosclerosis. The VSMCs are reported to be one of crucial elements in the progression of atherosclerosis [22,23] so the cellular proliferation and migration-related pathways are suggested as effective therapeutic targets [24]. The goal of this study was to investigate the effect of Heparin on the VSMC migration and its relation to NF-kB. Furthermore, the role of Heparin studied on the expression levels of some ECM genes involved in atherosclerosis such as collagen 1, and collagen 3. The role of Heparin was further clarified by incorporating Heparin to cellular cultures containing Betulinic acid and Ibrutinib.

Heparin derivatives and conjugates are reported to be explored as anti-thrombotic, alongside anticancer, wound healing, antiviral, and anti-inflammatory factors [25]. Furthermore, it is reported that Heparin influences angiogenesis, cell adhesion, migration, and proliferation of cells [26]. However, the effects of Heparin are dependent on some factors such as cell type, Heparin chemical structure, dosage, and exposure periods. Some studies showed that Heparin has an antiproliferative effect in stimulated cells, including vascular smooth muscle cells, stromal cells, embryonic stem cells, osteoclast cells, and tumor cells [12,[25], [26], [27], [28], [29], [30]]. Furthermore, the antiproliferative effects of Heparin are reported in a dose-dependent manner [31] so that the osteoblasts treated with Heparin did not show noticeable effects on the cell viability in the short time periods [32]. In contrast with the antiproliferative effects, some studies reported that Heparin increases the cell growth. Heparin might also be used in cell culture to increase the favorable activity of the growth supplements of human stem cells. Furthermore, Heparin has been shown to stimulate the Wnt and FGF signaling pathways in human embryonic stem cells (hESCs), thereby increasing their proliferative capacity. Another study showed that human VSMCs harvested from mature arteries exhibit the high growth rate and lifespan. In another study, Heparin sodium has been shown to enhance virtually hMSC proliferation [26,28,33,34]. The power of mitogens known as Heparin-binding growth factors (HBGFs) was also reported in human vascular smooth muscle cells [35]. Unfractionated Heparin (UFH) has been found to increase the osteogenic differentiation of human stromal cells resulting in osteoclastogenesis. The incorporation of UFH into culture medium including low doses of endothelial cell growth factor allowed the multiplication of cloned human endothelial cell strains [28]. Also, Heparin-bound EGF-like growth factor (HB-EGF) stimulated the tumor cell proliferation [36]. In addition, Heparin has some impacts on transcription factors. The nuclear factor kappa B is one of the transcription factors that might be impacted [13]. Heparin decreased the p65 level and caused the blocking of NF-kB in TNFα-induced fibroblasts like synoviocytes [37]. Furthermore, the VSMC proliferation reduced when NF-kB suppressed [38].

These studies showed that Heparin has a dual impact on cell growth dependent on the dosage, chemical structure and treatment periods. According to the different effects of Heparin on cellular proliferation, the different doses used in this study were based on the VSMC viability results. Since the lower Heparin doses (5 IU and 15 IU) did not affect the NF-kB expression levels thus Heparin 30 IU investigated on VSMC migration. The results showed the Heparin 30 IU increases significantly the VSMC migration in the 24-h and 48-h periods. Since Heparin 30 IU increased the NF-kB gene and protein expression levels thus these results in agreement with the above studies [38], suggested that NF-kB might be involved in the migration of VSMCs. Furthermore, the results showed that the role of Heparin 30 IU on the VSMC migration does not relate to the changes of collagen 1 and collagen 3. However, in contrast with some studies [37] that showed the Heparin suppresses NF-kB, its increase in this study may be due to the used dose, chemical structure and the failure of its incorporation with NF-kB inducers.

On the other hand, some reports showed that vascular smooth muscle cells are involved in the production of extracellular matrix (ECM) compounds [39]. Collagen 1 found highly around smooth muscle cells in atherosclerosis plaque. Collagen 3 discovered in dense deposits next to the elastic laminae and in diffuse intimal thickening. There were the reports on the reduction of collagen 3 with the progression of atherosclerosis. Additionally, collagen 3 is believed to make up to 70% in the cultured aortic smooth muscle cells [40,41]. Some studies showed that Heparan sulfate reduces the synthesis of collagen 1 in VSMCs via a reduction in the incorporation of thymidine in DNA [42]. Heparin (50 μg/ml) caused to remodel the ECM by changing GAG-deficient and collagen-rich patterns in the human VSMCs [34]. Heparin (100 μg/ml) and other polysulfates also reduced the collagen 1 in fibroblasts [31]. It was discovered that collagen levels were lower in the ECM of the intima of animals treated with Heparin [43]. The effect of Heparin was also reported to increase collagen synthesis. A study revealed that Heparin increased the synthesis of collagen in osteoblast-like cell line (MC3T3-E1) following 15 and 30 day periods [44]. Also, Heparin increased the collagen 1 precursors in cultures of rat vascular smooth muscle cells [45]. Furthermore, the collagen 1 synthesis related to Heparin doses and exposure periods. In agreement with this hypothesis, the study results showed that the Heparin 15 IU and 30 IU increase the collagen 1 expression level in the 48-h period while the lower dose (5 IU) had no significant effect on collagen 1 in VSMCs in both periods. In contrast with the collagen 1, the collagen 3 increased in the Heparin doses 5 IU and 15 IU in both 24 and 48-h periods. These results suggested that the Heparin 5 IU and 15 IU are safe for the function of VSMCs.

In order to evaluate the Heparin effects, the incorporation of Heparin was studied in the VSMC cultures including Betulinic acid and Ibrutinib in this study. There were controversies on the roles of Betulinic acid in the cell growth and NF-kB expression levels [14,[46], [47], [48], [49], [50], [51], [52], [53]]. These studies showed that the effects of Betulinic acid are dependent on the applied doses and exposure periods. Some reports showed Betulinic acid affects the collagen synthesis. For example, Betulinic acid restricted the collagen synthesis in cancerous cells [49]. Based on the cellular viability, the study results showed that Betulinic acid improves the VSMC migration with the increase of exposure periods. Since Betulinic acid inhibited the NF-kB gene and protein expression levels thus the study results suggested that the VSMC migration and proliferation are independent of NF-kB involved pathways. Furthermore, Betulinic acid inhibited collagen 1 and collagen 3 in the 24-h period. The incorporation of Heparin to the cultures including Betulinic acid increased the collagen 1 in a dose-dependent mode. Heparin (5 IU and 15 IU) also increased the collagen 3 level in 48-h period.

Ibrutinib is used in immunotherapy, chemoimmunotherapy, cell-based therapy, and other targeted therapies [54]. Ibrutinib, known as a powerful and selective inhibitor of Bruton's tyrosine kinase (BTK), has been demonstrated to decrease the cellular migration and proliferation [[55], [56], [57], [58], [59], [60], [61]]. Moreover, Ibrutinib has also been shown to increase apoptosis together with decreasing cell proliferation, adhesion, and migration [62]. Ibrutinib also inhibited the nuclear synthesis of the NF-kB and platelet aggregation [63]. In this study, the Ibrutinib was used in according to the VSMC viability. In agreement with the above studies, the study results showed that Ibrutinib inhibits the VSMC migration. It also suppressed the collagen 1, and collagen 3 gene expression levels in the 24-h period. Furthermore, Ibrutinib suppressed the NF-kB gene expression levels in both periods so that the data confirmed the previous reports [63] on the role of NF-kB in the suppression of cell migration. The incorporation of Heparin in cultures including Ibrutinib improved significantly the gene collagen 1 expression levels. However, the NF-kB protein values were not associated to NF-kB expression levels in the Heparin dose 30 IU incorporated to Ibrutinib. These results suggested that the physico-chemical characteristics of Heparin on binding small molecules such as Betulinic acid and Ibrutinib might affect their transport into the cytosol and finally, modification of cellular signaling pathways.

## 5 Conclusion

The study results showed that Heparin 30 IU increases the cell migration and the NF-kB gene expression levels, so we suggested the NF-kB axis might be involved in the migration of VSMCs. Furthermore, Heparin 5 IU and 15 IU were safe for VSMCs since there were no changes in the NF-kB expression levels while these doses increased the collagen 3 expression levels. The results also showed the Betulinic acid, but not Ibrutinib, increases the VSMC migration in the longer periods while both Betulinic acid and Ibrutinib decreased the NF-kB gene expression levels. The results suggested that the NF-kB axis might be involved in the VSMC migration by Ibrutinib. Based on the incorporation of Heparin into the cultures including Betulinic acid and Ibrutinib, the results suggested that the Heparin was not only involved directly in cellular signaling pathways but also affected the cellular messages of small molecules.

## 6 Ethics approval and consent to participate

Not applicable.

## 7 Human and animal rights

No animals/humans were used for this review.

## 8 Consent for publication

Not applicable.

## Availability of data and materials

The data generated and analyzed during the current study are available from the corresponding author on reasonable request.

## Funding

It is supported by Iran University of Medical Sciences (No. 22927).

## CRediT authorship contribution statement

**Nafiseh Shokri:** Writing – review & editing, Writing – original draft, Resources, Investigation, Data curation. **Ghasem Ghasempour:** Investigation. **Ali Akbar Soleimani:** Investigation. **Mohammad Elahimanesh:** Investigation. **Mohammad Najafi:** Supervision.

## Declaration of competing interest

NO.

## Data Availability

Data will be made available on request.
