# Supplementary material for: NF-kB affects migration of vascular smooth muscle cells after treatment with heparin and ibrutinib
Source: Biochem Biophys Rep. 2024 Mar 16;38:101685. doi: 10.1016/j.bbrep.2024.101685 (PMC10957380; doi:10.1016/j.bbrep.2024.101685)
Supplement: Multimedia component 1 [file mmc1.docx]

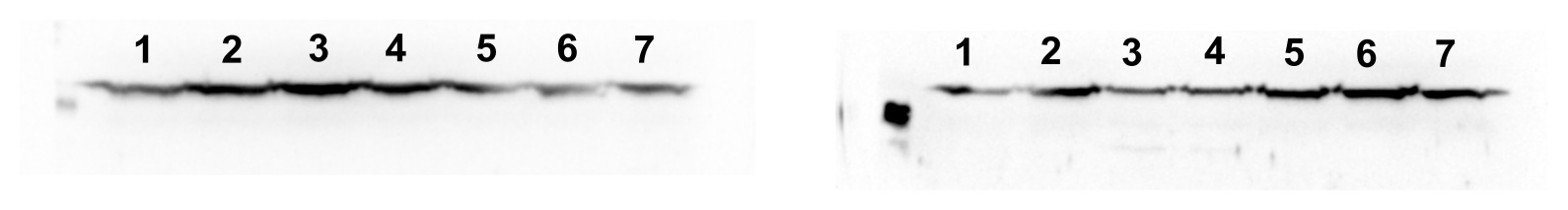


**C**: ß-actin protein expression levels in vascular smooth muscle cells (VSMCs) in the studied groups. **1**. Control, **2**.Heparin 30IU, **3**. Heparin 30IU + Betulinic acid 60µM, **4**. Betulinic acid 60µM, **5**. Control, **6**.Ibrutinib 2µM, **7**.Heparin 30IU + Ibrutinib 2µM . 48 hours treatment

.**A**: ß-actin protein expression levels in vascular smooth muscle cells (VSMCs) in the studied groups. **1**. Control, **2**.Heparin 30IU, **3**. Heparin 30IU + Betulinic acid 60µM, **4**. Betulinic acid 60µM, **5**. Control, **6**.Ibrutinib 2µM, **7**.Heparin 30IU + Ibrutinib 2µM . 24 hours treatment


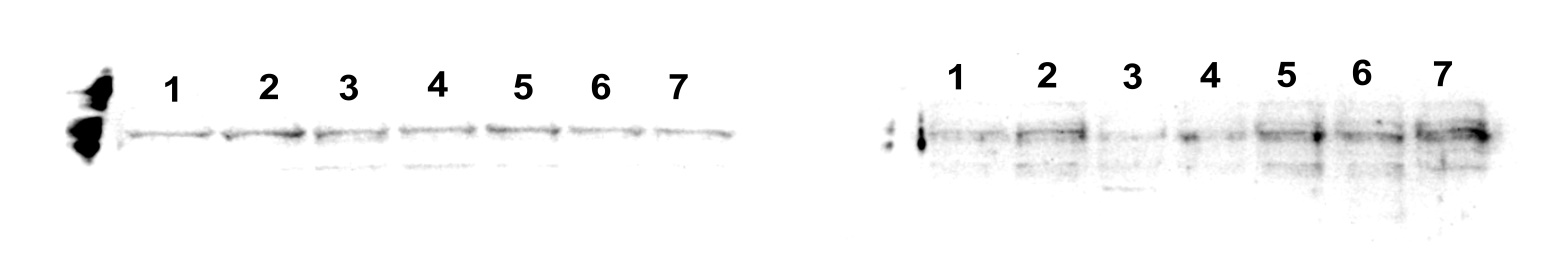


**B**: NF-kB protein expression levels in vascular smooth muscle cells (VSMCs) in the studied groups. **1**. Control, **2**.Heparin 30IU, **3**. Heparin 30IU + Betulinic acid 60µM, **4**. Betulinic acid 60µM, **5**. Control, **6**.Ibrutinib 2µM, **7**.Heparin 30IU + Ibrutinib 2µM . 24 hours treatment

**D**: NF-kB protein expression levels in vascular smooth muscle cells (VSMCs) in the studied groups.. **1**. Control, **2**.Heparin 30IU, **3**. Heparin 30IU + Betulinic acid 60µM, **4**. Betulinic acid 60µM, **5**. Control, **6**.Ibrutinib 2µM, **7**.Heparin 30IU + Ibrutinib 2µM . 48 hours treatment

Supplement 1: Uncropped western bolting images.
